# Characterization of the recombinant *Brettanomyces anomalus β*‐glucosidase and its potential for bioflavouring

**DOI:** 10.1111/jam.13200

**Published:** 2016-07-27

**Authors:** Y. Vervoort, B. Herrera‐Malaver, S. Mertens, V. Guadalupe Medina, J. Duitama, L. Michiels, G. Derdelinckx, K. Voordeckers, K.J. Verstrepen

**Affiliations:** ^1^ VIB Laboratory of Systems Biology Leuven Belgium; ^2^ CMPG Laboratory for Genetics and Genomics KU Leuven Leuven Belgium; ^3^ Leuven Food Science and Nutrition Research Centre Leuven Belgium

**Keywords:** *Brettanomyces*, enzyme, flavours, yeast, *β*‐glucosidase

## Abstract

**Aim:**

Plant materials used in the food industry contain up to five times more aromas bound to glucose (glucosides) than free, unbound aromas, making these bound aromas an unused flavouring potential. The aim of this study was to identify and purify a novel *β*‐glucosidase from *Brettanomyces* yeasts that are capable of releasing bound aromas present in various food products.

**Methods and Results:**

We screened 428 different yeast strains for *β*‐glucosidase activity and are the first to sequence the whole genome of two *Brettanomyces* yeasts (*Brettanomyces anomalus* and *Brettanomyces bruxellensis*) with exceptionally high *β*‐glucosidase activity. Heterologous expression and purification of the identified *B. anomalus β*‐glucosidase showed that it has an optimal activity at a higher pH (5·75) and lower temperature (37°C) than commercial *β*‐glucosidases. Adding this *B. anomalus β*‐glucosidase to cherry beers and forest fruit milks resulted in increased amounts of benzyl alcohol, eugenol, linalool and methyl salicylate compared to *Aspergillus niger* and Almond glucosidase.

**Conclusions:**

The newly identified *B. anomalus β*‐glucosidase offers new possibilities for food bioflavouring.

**Significance and Impact of the Study:**

This study is the first to sequence the *B. anomalus* genome and to identify the *β*‐glucosidase‐encoding genes of two *Brettanomyces* species, and reports a new bioflavouring enzyme.

## Introduction

Flavour is considered to be one of the major quality attributes of a food product and flavour compounds represent over a quarter of the world market for food additives (Mouret *et al*. 2015). Due to an increased environmental awareness, nowadays consumers prefer naturally produced flavours, obtained from plant materials or by fermentation, where micro‐organisms add specific flavours, over chemically synthesized aroma compounds (Vanderhaegen *et al*. 2003). Although plant material contains volatile flavours, it also contains two‐ to five times as many nonvolatile aroma molecules that are bound to monosaccharides like *β*‐glucose (i.e. *β*‐glucoside) or more rarely disaccharides (Sarry and Günata 2004). These nonvolatile aromas or ‘aglycones’ can contribute to the food aroma when they are released from the sugar molecule (glycone). This release can occur through acidic or enzymatic hydrolysis (Li *et al*. 2013). Since acidic hydrolysis occurs at very low pH and induces aglycone rearrangements (Gunata *et al*. 1988), enzymatic hydrolysis by *β*‐glucosidases is the preferred industrial strategy to release aglycones. *β*‐Glucosidases are often added during food production because (i) the ingredients or micro‐organisms present lack or show little *β*‐glucosidase activity, (ii) because of glucose or ethanol inhibition of present glucosidases or (iii) because enzymatic activity of present glucosidases is inhibited by the pH or temperature of the application (Gil *et al*. 2005). Various *β*‐glucosidases from moulds (Thongpoo *et al*. 2014), bacteria (Michlmayr *et al*. 2009) and yeasts (Wang *et al*. 2012) already have been characterized. Additionally, heterologous expression systems allow producing higher levels of *β*‐glucosidases (Zietsman *et al*. 2010). However, some current commercial *β*‐glucosidases show unwanted side activities. This is for example the case for AR2000, a cell extract from *Aspergillus niger* that is used to enhance the aroma of wines. Apart from releasing aglycones, AR2000 also hydrolyses anthocyanins, causing decolourization of red wines (Sarry and Günata 2004). Hence, there is a need for *β*‐glucosidases that perform well under the conditions of the production process.

*Saccharomyces* yeasts are often used in food production and generally release desirable aroma compounds, such as esters (Steensels *et al*. 2014), but show only weak and variable *β*‐glucosidase activity (Rosi *et al*. 1994). More pronounced *β*‐glucosidase activities are mainly found in non‐*Saccharomyces* yeasts such as *Brettanomyces*,* Candida*,* Debaromyces* and *Kloeckera* (Wu *et al*. 2014). While commonly known as spoilage organisms in wine, *Brettanomyces* are also part of the natural, desirable microbiome in the production of lambic and gueuze beers and wines like Château de Beaucastel (Steensels *et al*. 2015). Although *Brettanomyces* yeasts have higher *β*‐glucosidase activity than *Saccharomyces* yeasts, no genetic information concerning the genes encoding *β*‐glucosidases in these species is available (Woolfit *et al*. 2007; Crauwels *et al*. 2014; Steensels *et al*. 2015).

In this study, we identified the *β*‐glucosidase‐encoding genes in *Brettanomyces anomalus* by high‐quality whole‐genome sequencing. The *β*‐glucosidase was heterologously expressed, purified and thoroughly characterized. We found that the *B. anomalus β*‐glucosidase performs optimal at pH 5·75 and 37°C, showing higher activity than commercial enzymes. Moreover, we found significant aroma differences in cherry beers and fruit milk beverages treated with the *B. anomalus* or commercial *β* ‐glucosidases.

## Materials and methods

### Reagents and enzymes

The following products were purchased from Sigma Aldrich (Diegem, Belgium): 2‐meraptoethanol (99%), almond *β*‐glucosidase (6·6 U mg^−1^), amygdalin (>99%), arbutin (>98%), citric acid monohydrate (>98%), d‐glucose monohydrate (>98%), imidazole (>99%), Luria‐Bertani (LB) medium, magnesium acetate tetrahydrate (>99%), PBS (10× concentrate), salicin (>99%), SDS and sorbitol (>98%). Ammonium acetate (>98%), EDTA, ethanol, HCl (37%), sodium acetate trihydrate, sodium chloride, disodium hydrogen phosphate dihydrate (>99%) and sodium hydroxide (99·5%) were purchased from VWR (Heverlee, Belgium). Other products used in this study include 4‐nitrophenol (Fluka, Diegem, Belgium), agar (Invitrogen, Ghent, Belgium), amino acid mix with (NH_4_)_2_SO_4_ (MP Biomedicals, Brussels, Belgium), AR2000 (*A. niger* cell extract showing glucosidase activity; DSM, Brussels, Belgium), bactopeptone (BD Bioscience, Erembodegem, Belgium), cellobiose (>98%; Fluka, Diegem, Belgium), DNaseI (Roche, Vilvoorde, Belgium), glycerol (99%; Biosolve BV, Valkenswaard, Netherlands), Glucose Oxidase‐ Phenol 4‐Aminoantipyrine Peroxidase (GOD‐PAP) (Dialab, Belsele, Belgium), hop pellets (Saaz Saaz), Isopropyl β‐D‐1‐thiogalactopyranoside (IPTG; >99%; Biosolve BV, Valkenswaard, Netherlands) isomerized hop extract (Brewferm, Beverlo, Belgium), isopropanol (>99·8%, Labscan, Zedelgem, Belgium), kanamycin sulphate (Gibco, Ghent, Belgium), malt extract light (7–12 EBC; Brouwland, Beverlo, Belgium), protease inhibitor cocktail (Roche), RNase (Westburg), sour cherry extract (Alcoferm, Beverlo, Belgium), Tris Base (Formedium, Hunstanton, UK), yeast extract (LabM, Brussels, Belgium), yeast nitrogen base (YNB; MP Biomedicals, Brussels, Belgium) and zymolyase (AMSBIO, Abingdon, UK).

### Screening of yeast collection for *β*‐glucosidase activity and ethanol sensitivity

The *β*‐glucosidase activity of 428 yeast strains from different fermentation industries (Table S1) was assessed by spotting assays on agar plates with a *β*‐glucoside (cellobiose, salicin or arbutin; Table S2) as carbon source. Spottings on 2% glucose and 2% ethanol plates were added as growth control. Lab strain BY4741 (Brachmann *et al*. 1998) was used as negative control as it does not show any *β*‐glucosidase activity. Single colonies from YPD plates were grown overnight in 600 *μ*l yeast extract peptone dextrose (YPD) at 30°C and 200 rev min^−1^. OD_600_ was measured, cultures were diluted to OD 1 and strains were spotted on the agar plates. Growth was evaluated by checking colony size after 48 h of incubation (72 h for non*Saccharomyces*) at 30°C.

### Whole‐genome sequencing of YV396 and YV397 and genome comparison

Genomic DNA of YV396 (*B. anomalus*) and YV397 (*Brettanomyces* *bruxellensis*) was sequenced by the Beijing Genome Institute (BGI) using paired‐end Illumina HiSeq sequencing. One paired‐end library with 86 bp read length and 500 bp insert size and two mate pair libraries with 49 bp read length and 2000 and 5000 bp insert sizes were developed for each sample. Initial average coverages for each library were 127×, 40× and 40× respectively. Before *de novo* sequencing, clean reads were obtained using the following filters: (i) Remove reads with more than 10% ‘N’ basepairs; (ii) remove reads with more than 20 bp with low quality (<20); (iii) Remove reads showing an overlap of at least 15 bp with the adapter sequence; (iv) Remove the first 4 bp of each read. Because the 2000 bp and 5000 bp libraries are obtained by DNA circularization, the following additional filters were applied for these libraries: (i) Remove reads with significant poly‐A structure and (ii) Remove reads with k‐mer frequency equal to 1. *De novo* assembly was performed using soapdenovo software. YV396 was assembled in 30 scaffolds with an N50 value of 1·55 Mbp and a total assembly size 12·88 Mbp. YV397 was assembled in 85 scaffolds with an N50 of 732 Kbp and a total assembly size of 13·06 Mbp (Crauwels *et al*. 2014). The software genmark‐es (Ter‐Hovhannisyan *et al*. 2008) was used to perform gene prediction on the scaffolds obtained with soapdenovo. blastp searches were performed on the predicted genes to identify potential orthologs of *β*‐glucosidase genes in the genes annotated using genmark‐es. Sequences were compared with known *β*‐glucosidase genes from *Aspergillus aculeatus* (Genbank Acc. No. JN121996.1), *Kluyveromyces marxianus* (Genbank Acc. No. X05918.1) and *Trichoderma reesei* (Genbank Acc. No. XP_006969215.1) using clustalw to identify the *β*‐glucosidase‐encoding genes of YV396 and YV397.

### Construction of synthetic DNA fragments

Synthetic DNA fragments containing the *β*‐glucosidase gene of YV396 (*B. anomalus*), YV397 (*B. bruxellensis*) or YV404 (*K. marxianus*), a purification (Lys_6_) and immobilization tag (His_6_), the Shine‐Dalgarno sequence, a XbaI and XhoI recognition sequence and two linkers that allow using the enzyme without His_6_ and/or Lys_6_ tag (Fig. S1) were synthesized by Geneart. The constructs were codon optimized (Fig. S2) for expression in *Escherichia coli*.

### Production of the *Brettanomyces anomalus* (YV396) *β*‐glucosidase

**Figure 1:**
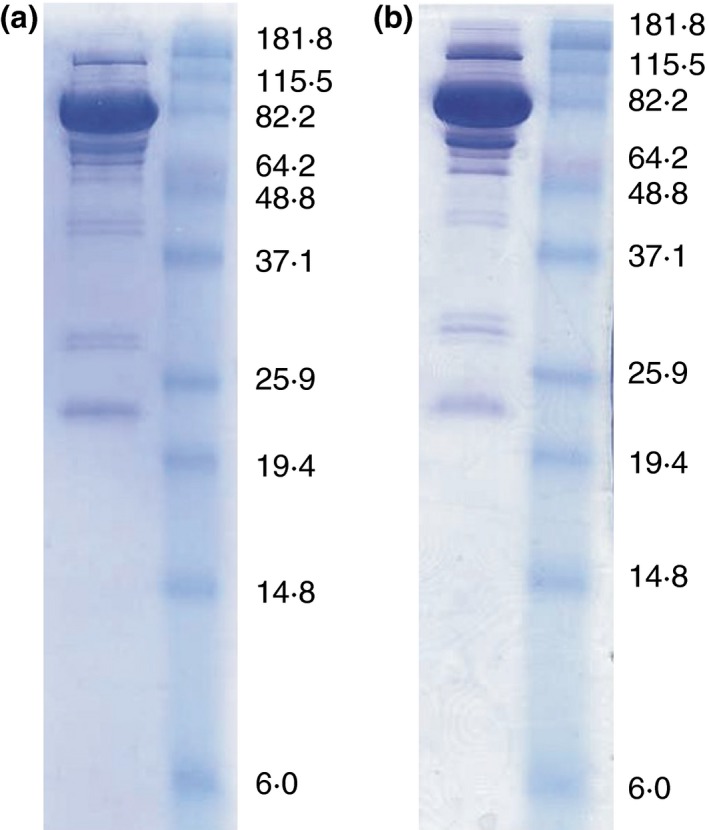
SDS‐PAGE of two produced purified protein fractions. The purity of two batches of isolated *Brettanomyces anomalus* enzyme was assessed by loading 10 *μ*g of product for SDS‐PAGE in reducing conditions. The biggest band on the gel corresponds to the glucosidase enzyme construct with a molecular weight of 96·342 kDa. Additional bands observed on the SDS‐PAGE gel indicate degradation of the isolated protein (confirmed by Western blot analyses). Protein concentrations were measured using BCA method with BSA as standard and yielded a concentration of 1·68 mg ml^−1^ (a) and 1·60 mg ml^−1^ (b).

*Escherichia coli* strain BL21 (DE3) was transformed with pET28‐YV396‐His_6_. Bacteria were grown overnight at 37°C in LB‐kanamycin (50 *μ*g ml^−1^) before 100× dilution in a 20 l fermenter with LB‐kanamycin (50 *μ*g ml^−1^) and 1% glycerol. The initial stirring and airflow were respectively 200 rev min^−1^ and 1·5 l min^−1^. Further, flow was automatically adapted to keep pO_2_ at 30% and temperature at 37°C until cells were grown to OD 0·8. Expression was induced by adding 0·5 mmol l^−1^ IPTG overnight at 20°C and mostly occurred in the cytoplasm and, to lesser extent, in inclusion bodies (see Fig. S3). Cells were harvested and frozen at −20°C. Two independent fermentations yielded 240 and 229 g of cells. After thawing, cells were resuspended at 3 g ml^−1^ in 20 mmol l^−1^ NaH_2_PO_4_ pH 7·4, 500 mmol l^−1^ NaCl, 20 mmol l^−1^ imidazole, one tablet 100 ml^−1^ protease inhibitor cocktail and 1 mg 100 ml^−1^ DNaseI. Cells were sonicated (Sonic Ultra Cell with 13 mm probe, Sonics & Materials, Newtown, CT, USA) on ice at 70% amplitude for 24 cycles of 5 s with 9 s between different cycles. The cytoplasmic suspension was centrifuged at 18 000 ***g*** for 30 min at 4°C. Next, the supernatant was applied to the Äkta purification system equipped with a 1 ml Ni‐Sepharose 6 FF chromatography column (GE Healthcare, Diegem, Belgium). In this column, the histidine tag of the protein will interact with Ni^2+^ ions as described by Ueda *et al*. (2003). The column was equilibrated with 20 mmol l^−1^ NaH_2_PO_4_ pH 7·4, 500 mmol l^−1^ NaCl, 20 mmol l^−1^ imidazole to base line, washed with 10 column volumes of 20 mmol l^−1^ NaH_2_PO_4_ pH 7·4, 20 mmol l^−1^ NaCl, 400 mmol l^−1^, 50 mmol l^−1^ of imidazole and eluted in 10 column volumes of 400 mmol l^−1^ imidazole in the same buffer. All flow rates were 1 ml min^−1^. Detection was done at 280 nm. The protein of interest eluted in the 400 mmol l^−1^ imidazole fraction in a rather broad peak spread over the 10 column volumes. The elution fraction from the Ni‐Sepharose 6FF column was injected on a Superdex 200 XK26 × 65 column with PBS as running solution (4 ml min^−1^) for formulation and to remove minor contaminants. The obtained purified fractions were analysed by SDS‐PAGE in reducing conditions (Fig. 1) and protein concentration was measured using the Micro‐BCA assay with BSA standard. The two independent purifications yielded 47 and 48 mg of protein in 28 (1·68 mg ml^−1^) and 30 ml (1·6 mg ml^−1^).

### Optimal pH and temperature, thermostability and apparent *K*_M_ and *k*_cat_ values of the different enzymes

Optimal pH and temperature, thermostability and apparent *K*_M_ and *k*_cat_ values were determined using an enzymatic assay that was performed in the linear range of product formation (Fig. S4) in 96 well PCR blocks (Bio‐Rad, Nazareth, Belgium; C1000^™^ Thermal Cycler). Twenty‐five microlitres of McIlvaine buffer (0·1 mol l^−1^ citric acid, 0·2 mol l^−1^ Na_2_HPO_4_, pH specific), 15 *μ*l substrate solution [25 mmol l^−1^ cellobiose, amygdalin or salicin in McIlvaine buffer (pH specific and various concentrations)] and 10 *μ*l enzyme solution (3·78*10^−2^ and 1·89*10^−2^ *μ*mol l^−1^) or 10 *μ*l McIlvaine buffer (blank) were mixed and incubated for 1 h at specific temperature, after which a 10 min denaturation step at 95°C was applied. Next, 30 *μ*l McIlvaine buffer was added. Fifty microlitres of sample was added to 100 *μ*l of GOD–PAP, incubated for 15 min at 30°C and 200 rev min^−1^ and absorbance was measured at 505 nm.

Optimal pH and temperature were determined by performing this assay for cellobiose at pH ranging from 3 to 7 at 30°C and temperatures ranging from 15 to 70°C at pH 5 respectively. We determined the amount of cellobiose that was hydrolysed per minute, which was normalized to the condition with the highest activity value.

Thermostability of the *β*‐glucosidases was measured by incubating the enzymes 4 h in PBS at 4, 30, 40, 50, 60, 70 or 80°C after which the enzymatic assay was performed for cellobiose at 30°C and pH 5. Enzymatic activity of the heat‐treated enzymes was normalized to the untreated samples (incubation at 4°C).

*K*_M_ and *k*_cat_ values were measured for cellobiose, amygdalin and salicin (0·15–60 mmol l^−1^) at pH 4·5 and 17°C and pH 5·75 and 37°C. Equimolar amounts of *B. anomalus* and almond *β*‐glucosidase and the average mass amount of *B. anomalus* and almond *β*‐glucosidase for AR2000 were used, considering this is a crude cell extract. Enzymatic activity was calculated as the amount of glycoside hydrolysed per minute, *k*_cat_ values as enzymatic activity per enzyme concentration. Data were analysed using graphpad prism 6.01 (La Jolla, CA, USA).

### Industrial applications

The different *β*‐glucosidases were tested in beer fermentations and fruit milk beverages. For beer samples, 600 ml 12°P malt with 4 ml isomerized hop extract or cherry extract or 400 mg hop pellets were pitched with 10^7^ cells YV15 (*β*‐glucosidase negative ale strain, Table S3), per ml and 10·5 nmol *B. anomalus* or almond *β*‐glucosidase or the average weight amount of protein of AR2000. Beers were fermented for 7 days at 18°C. Fruit milk beverages were prepared by defrosting, mixing and centrifuging frozen forest or tropical fruit. Next, 180 ml of ultra‐high temperature processed (UHT) milk was mixed with 20 ml pasteurized juice (12 min at 80°C) and 3·5 nmol *B. anomalus* or almond *β*‐glucosidase or the average weight amount of protein of AR2000 and incubated for 24 h at 37°C.

### Sensory analysis

The aroma of beers and milk beverages was evaluated by a test panel consisting of 20 untrained persons. Samples with *B. anomalus β*‐glucosidase were compared to samples with AR2000, almond or without enzyme. Differences were significant when 55% of the panelists indicated the same sample as being different (*α *= 0·05, *β *= 0·10 and *p*_d_ = 50%), and next, these samples were subjected to headspace‐solid phase microextraction‐gas chromatography‐mass spectrometry (HS‐SPME‐GC‐MS) and preference tests. In preference tests, panelists had to indicate their preference for the aroma of samples with *B. anomalus β*‐glucosidase or samples with AR2000, almond or without enzyme. A significant preference was noted when at least 75% of the panelists preferred the same sample (*α *= 0·05, *β *= 0·40 and *p*(preference for a specific aroma) = 75%) (Meilgaard *et al*. 2007).

### Identification and quantification of volatile compounds in beverages

Aroma compounds of beverages with significant differences were identified and quantified by HS‐SPME GC‐MS. A 20 ml vial with 5 ml of sample, 1·75 g NaCl and 5 *μ*l of internal standard (IS; 2‐heptanol, 250 *μ*g ml^−1^) was immersed in a 40°C water bath. After 5 min of equilibration, a triphase DVB/Carboxen/PDMS 50/30 *μ*m SPME fibre (Supelco Co., Bellefonte, PA) was exposed to the headspace for 30 min. Next, volatiles were desorbed in the GC‐MS (Shimadzu, Brussels, Belgium; QP2010 Ultra Plus) by heating for 5 min at 250°C. The GC‐MS was equipped with a HP‐5 ms nonpolar column (Agilent, Diegem, Belgium; 30 m × 0·25 mm i.d., 0·25 *μ*m thin layer), helium was used as carrier gas at a pressure of 100 kPa and samples were injected in split/splitless mode. The temperature program is displayed in Fig. S5. The mass detector was operated in scan mode (35–500 amu) using electronic impact ionization (70 eV). The interface and detector were kept at 250°C.

Linear n‐alkanes (C8 to C19) were injected in the GC‐MS as external retention index (RI) markers. RI of aglycones were calculated using cubic spline interpolation. Compounds were identified using amdis ver. 2.71, followed by matching deconvoluted spectra to commercial GC/MS libraries described in Robert and Adams (2007) and Mondello (2011). RI of aglycone standards were used to confirm the identification. Since some compounds coeluted, analysis was performed by integrating over characteristic ions using openchrom (ver. 0.9.0). Next, integrated areas were converted back to a total ion scale based on the relative abundance of each characteristic ion in the spectrum of each compound. Relative concentrations were calculated by normalizing to the 2‐heptanol IS peak and comparing the log‐transformed relative peak area of each compound across treatments using a linear mixed model analysis, in which treatment and biological replicate nested within treatment were coded as fixed and random factors. Treatment averages were compared in a pairwise fashion using Tukey's post hoc tests and final significance levels were corrected for multiple testing across all compounds using the Bonferroni procedure with r package lme4. In addition, we calculated the absolute concentration (in ppb) of aglycones based on their measured response factor relative to the IS.

## Results

### Screening of yeast strains

In order to identify yeast strains with high *β*‐glucosidase activity, a high‐throughput screen of 428 strains (Table S1) was performed doing spotting assays. Forty‐five percentage of the *Saccharomyces* strains and 47% of the non*Saccharomyces* yeasts grew on all *β*‐glucoside media. Two *Brettanomyces* strains that were isolated from spontaneous beer fermentations (YV396; *B. anomalus* and YV397; *B. bruxellensis*) had pronounced growth on *β*‐glucoside and ethanol media (Table S3). *Kluyveromyces marxianus* (YV404), a species with known *β*‐glucosidase activity (Yoshida *et al*. 2009), grew on *β*‐glucoside media but very poorly on ethanol. Based on these results, we isolated gDNA of the two *Brettanomyces* strains for whole‐genome sequencing to identify potential *β*‐glucosidase‐encoding genes.

### Whole‐genome sequencing and construction of synthetic DNA fragments

The assembled high‐coverage genome sequences of *B. bruxellensis* (YV397) and *B. anomalus* (YV396) and their *β*‐glucosidase‐encoding genes (with a gene size of 2526 and 2523 bp respectively) are freely available from Genbank under accession numbers PRJNA244003, PRJNA281311, KR181959 and KR181960 respectively. The *Brettanomyces β*‐glucosidases showed most similarity to the *K. marxianus* GH3 *β*‐glucosidase (Fig. S6). We identified several conserved amino acid regions, including a typical FGYGLSY domain, and predicted catalytic residues (aspartic acid and glutamic acid; Henrissat 1991; Sarry and Günata 2004; Quatrini *et al*. 2008). Based on several Swiss‐Models (ExPASy, Swiss Institute of Bioinformatics, Lausanne, Switzerland), the isolated *Brettanomyces β*‐glucosidases are probably monomeric.

The codon‐optimized DNA constructs containing the YV396, YV397 or *K. marxianus* GH3 *β*‐glucosidase (Fig. S1) were expressed in *E. coli* strain BL21(DE3) and purified on a Talon Metal Affinity Resin column (Clonetech, Leusden, Netherlands). Enzymatic activity was assessed on arbutin, salicin and cellobiose. Since the YV396 protein showed the highest specific activity (data not shown), large scale heterologous production and further experiments focused on this enzyme.

### Optimal pH and temperature and thermostability of *β*‐glucosidase enzymes

After large scale production of the YV396 *β*‐glucosidase, we determined optimal pH and temperature, thermostability and apparent *K*_M_ and *k*_cat_ values of the *B*. *anomalus β*‐glucosidase, and compared it to the commercially available almond GH1 and *A. niger* (AR2000) GH3 *β*‐glucosidases (Pozzo *et al*. 2010; Zhao *et al*. 2013).

**Figure 2:**
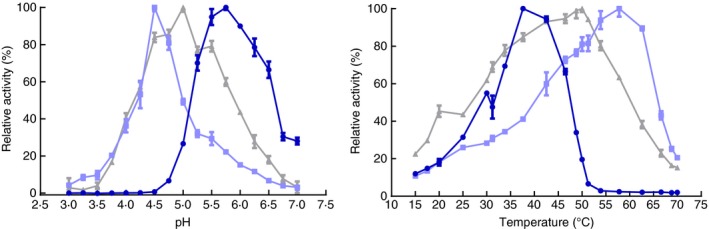
Effect of pH and temperature on the *β*‐glucosidase activity of the different enzymes on cellobiose (25 mmol l^−1^ in McIlvaine buffer). The optimal pH for AR2000 (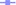), almond (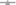) and *Brettanomyces anomalus* (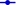) *β*‐glucsidase was 4·5, 5·0 and 5·75 respectively. The optimal temperature also differs between the three enzymes: AR2000, almond and *B. anomalus* enzyme showed maximal activity at 58°C, 50°C and 37°C respectively.

**Figure 3:**
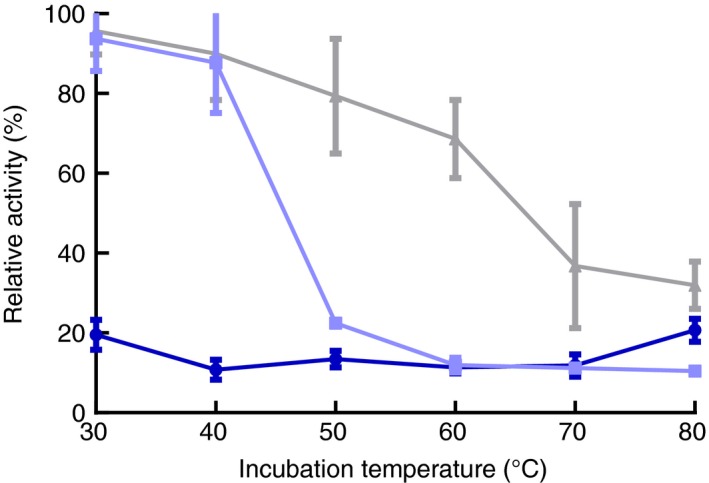
Thermostability of *β*‐glucosidases. Values are normalized to the untreated samples. Although the *Brettanomyces anomalus glucosidase* (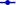) had lower relative thermal stability than almond (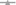) glucosidase and AR2000 (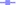), its absolute enzymatic activity was higher than all the values for Almond glucosidase and, when incubated at a temperature higher than 50°C; the values for AR2000 (Fig. S7).

The *β*‐glucosidases showed striking differences in their optimal pH and temperatures (Fig. 2). Optimal activity for *A. niger* (AR2000, used in wine industry), almond and *B. anomalus β*‐glucosidase was reached at pH 4·5, 5·0 and 5·75 and at 58°C, 50°C and 37°C respectively. Also their thermostability differed: the *B. anomalus β*‐glucosidase showed very low relative thermostability compared to both AR2000 and Almond glucosidase, although its absolute enzymatic activity was higher than Almond glucosidase and, when incubated at temperatures exceeding 50°C, higher than AR2000 (Fig. 3 and Fig. S7). Further enzymatic engineering can be useful to increase the thermostability and thus enzymatic activity of the *B. anomalus* glucosidase.

### Apparent *K*_M_ and *k*_cat_ values of the *β*‐glucosidases

*K*_M_ and *k*_cat_ parameters describe the enzyme's affinity for a substrate and the amount of substrate each enzyme site can convert per unit of time respectively. We determined these parameters for the different enzymes (*B. anomalus*, AR2000 and almond) for the substrates cellobiose, amygdalin and salicin at pH 4·5 at 17°C (simulating the end of beer fermentations) and pH 5·75 at 37°C (optimal conditions for the *B. anomalus β*‐glucosidase). We report *k*_cat_ and *K*_M_ as apparent values since the purity of enzyme solutions used is below 100%. Enzymatic activity plots and a summary of the apparent *K*_M_ and *k*_cat_ values are shown in Fig. S8 and Table 1. All enzymes followed Michaelis–Menten kinetics. AR2000 showed low *K*_M_ values, indicating its high affinity for *β*‐glucosides. Measured *k*_cat_ values confirmed that the *B. anomalus* enzyme works optimal at pH 5·75 and 37°C. The almond glucosidase had particularly high *k*_cat_ values for amygdalin, a glycoside commonly found in *Rosaceae*, proving that this enzyme is very suitable to hydrolyse these glycosides. The almond *β*‐glucosidase showed high *K*_M_ values for amygdalin and salicin as well.

**Table 1:** Kinetic parameters of different *β*‐glucosidases measured on different substrates, pHs and temperatures. Apparent *K*_M_ and *k*_cat_ values (*n* = 2) were determined excluding data points that showed lower enzymatic activity values because of product inhibition, caused by glucose accumulation (Fig. S8)

Substrate	Enzyme	pH 4·5 17°C		pH 5·75 37°C	
		*K*_M_ (mmol l^−1^)	*k*_cat_ (s^−1^)^a^	*K*_M_ (mmol l^−1^)	*k*_cat_ (s^−1^)^a^
Cellobiose	AR2000	0·60 ± 0·05	1·09 ± 0·04	0·52 ± 0·09	1·01 ± 0·09
	Almond	0·18 ± 0·06	0·05 ± 0·01	0·34 ± 0·08	0·06 ± 0·02
	*Brettanomyces anomalus*	1·15 ± 0·16	0·07 ± 0·01	15·06 ± 0·73	2·73 ± 0·32
Amygdalin	AR2000	0·40 ± 0·05	0·93 ± 0·04	0·12 ± 0·03	0·93 ± 0·16
	Almond	10·30 ± 0·09	1·11 ± 0·11	10·08 ± 0·13	3·45 ± 0·56
	*B. anomalus*	0·09 ± 0·03	0·07 ± 0·02	1·00 ± 0·05	0·31 ± 0·002
Salicin	AR2000	0·19 ± 0·06	1·08 ± 0·14	0·19 ± 0·06	0·86 ± 0·08
	Almond	10·95 ± 2·00	0·43 ± 0·01	19·65 ± 2·06	0·68 ± 0·01
	*B. anomalus*	6·49 ± 1·11	0·72 ± 0·02	1·01 ± 0·18	4·21 ± 0·34

a *k*_cat_ values for AR2000 are expressed in (*μ*mol substrate s^−1^ g_enzyme_^−1^) as AR2000 is a crude cell extract.

### Triangle tests of beers and milk beverages treated with *β*‐glucosidase enzymes

In a next step, the *β*‐glucosidases were added to beer fermentations (with hop or cherries) and tropical or forest fruit milk beverages in order to assess their effect on the aroma of these beverages. These beverages were chosen as their pH (4·5–5·2) was close to the pH optimum of the *B. anomalus* glucosidase. After production, a test panel evaluated the aroma of the beverages using triangle tests. We found significant differences between cherry beers with the *B. anomalus* enzyme and cherry beers with AR2000 or without enzyme. Also, differences were found between hopped beers with *B. anomalus β*‐glucosidase and without enzyme (Table 2). However, the hop extract was made by CO_2_ extraction of hop pellets which may lead to lower concentrations of glycosides in the hop extract, since glycosides are water‐soluble (Sarry and Günata 2004). Beers with hop pellets and different enzymes did not show any difference. Among the milk beverages, only forest fruit milks with different enzyme treatments were significantly different (Table 2). Given these results, cherry beers and forest fruit milk beverages were analysed with HS‐SPME GC‐MS and preference tests.

**Table 2:** Triangle tests of different beers and fruit milk beverages. Beers with isomerized hop extract (B + HE), cherry extract (B + CE) or hop pellets (B + HP) and milk beverages with tropical (M + T) – and forest fruit juice (M + F) were subjected to triangle tests by 20 untrained panelists. Cherry beers and milk beverages with forest fruit juice showed significant differences across the enzyme treatments. Beer with hop extract and *Brettanomyces anomalus β*‐glucosidase was also significant different from the untreated beer

Comparison	% correct identifications				
	Beers			Milk beverages	
	B** + **HE	B** + **CE	B** + **HP	M** + **T	M** + **F
*Brettanomyces anomalus* enzyme *vs* no enzyme	55^a^	55^a^	40	50	75^a^
*B. anomalus vs* almond *β*‐glucosidase	35	40	30	30	75^a^
*B. anomalus β*‐glucosidase *vs* AR2000	20	60^a^	40	50	75^a^

a Significant differences were observed between treatments at *α *= 0·05, *β *= 0·10 and *p*_d_ (the ability of a person to smell a certain difference) = 50% (Meilgaard *et al*. 2007).

### Volatile compounds released by *β*‐glucosidase enzymes in cherry beers and forest fruit milk beverages

**Figure 4:**
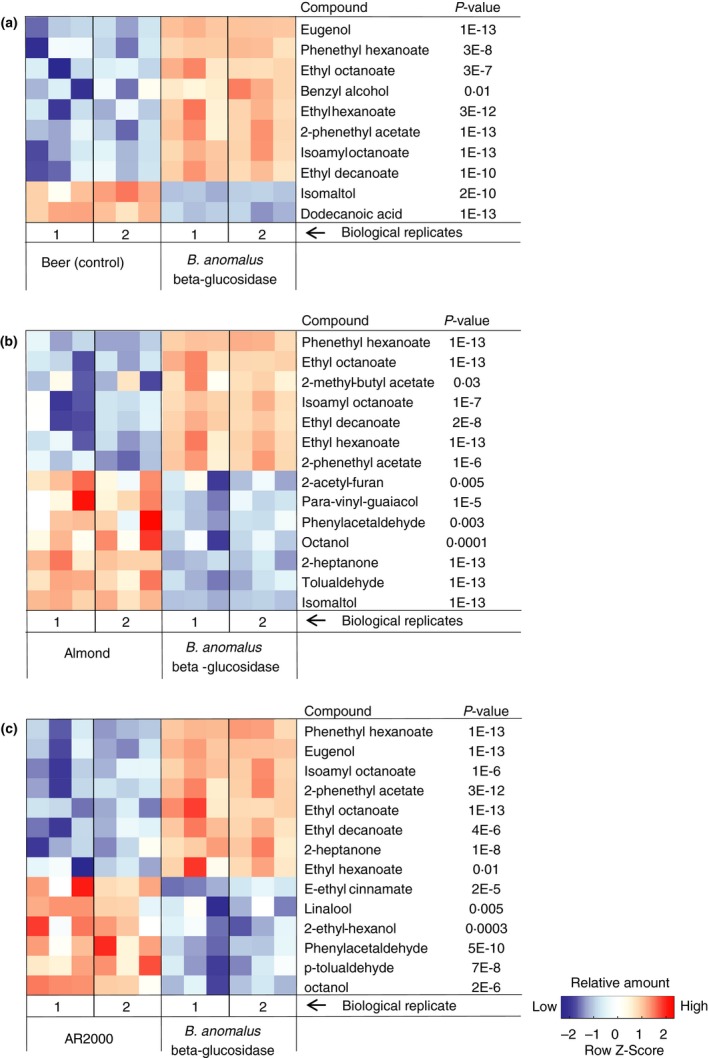
Volatile compounds in cherry beers with different enzyme treatments (*n* = 6). The aroma of cherry beers treated with the *Brettanomyces anomalus β*‐glucosidase differed significantly from beers without enzyme (a), with Almond *β*‐glucosidase (b) or AR2000 (c). Colour codes indicate relative concentrations, which were calculated as *z*‐scores of log‐transformed peak areas normalized relative to the internal standard (IS) peak. Significance levels were based on Tukey's post hoc tests and linear mixed model analysis on log‐transformed relative peak areas, in which biological replicate and treatment were coded as random and fixed factors. All *P*‐values are Bonferroni adjusted to correct for multiple testing across all 44 integrated compounds.

**Figure 5:**
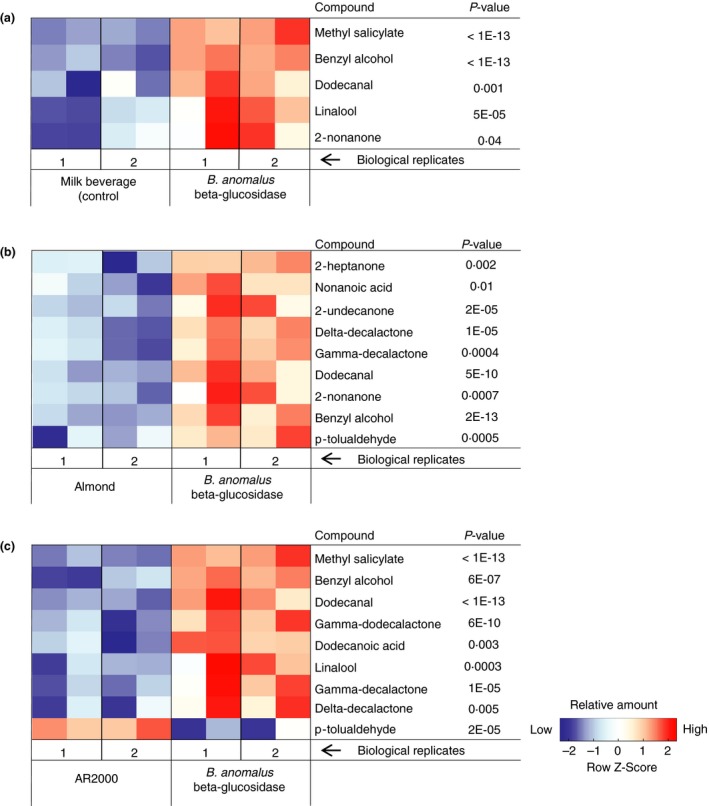
Volatile compounds in fruit milk beverages with different enzyme treatments (*n* = 4). The aroma of milk beverages treated with the *Brettanomyces anomalus β*‐glucosidase differed significantly from fruit milk beverage without enzyme (a), with Almond *β*‐glucosidase (b) or AR2000 (c). Colour codes indicate relative concentrations, which were calculated as described in Fig. 4.

HS‐SPME‐GC‐MS results of cherry beers and forest fruit milks are shown in Figs 4 and 5. Although many volatile compounds were found, we focused our analyses on relative changes in the amount of aglycones.

Beers with *B. anomalus β*‐glucosidase contained more eugenol (clove, honey aroma) and benzyl alcohol (sweet, flower) than untreated beers. Also, the *B. anomalus* glucosidase released more eugenol and less linalool (citral, flower; Mosciano 1994, accessed 13th January 2015) than AR2000. When looking at absolute concentrations of different aglycones (Table 3), it is clear that benzaldehyde (almond, cherry), linalool, eugenol, beta‐damascenone (honey, apple, peach) and geraniol (rose, lemon, flower) for all enzymes exceeded their odour threshold concentration (Meilgaard 1975; Acree and Heinrich 2014, accessed 12th October 2014).

**Table 3:** Absolute concentrations of aglycones (ppb) present in cherry beers (*n* = 6) and forest fruit milk beverages (*n* = 4) treated with different glucosidases. Values were determined using response factors relative to the 2‐heptanol internal standard (IS). Standard normal confidence intervals were calculated on a log scale and backtransformed to the original scale. All compounds, except benzyl alcohol (all samples) and methyl salicylate (fruit milk beverage with Almond *β*‐glucosidase) exceeded their odour threshold

Aglycone (ppb) (mean ± [CI])	No enzyme (control)	*Brettanomyces anomalus β*‐glucosidase	Almond *β*‐glucosidase	AR2000	Odor threshold (ppb)
Cherry beers					
Benzaldehyde	16 467 (10 865, 24 958)	22 107 (18 757, 26 055)	28 475 (26 822, 30 229)	23 445 (22 036, 24 944)	2000 (Meilgaard 1975)
Benzyl alcohol	247 (161, 379)	699 (494, 990)	948 (927, 971)	408 (351, 475)	900 000 (Meilgaard 1975)
Linalool	179 (130, 246)	161 (160, 162)	201 (175, 230)	246 (201, 302)	8–80 (Schönberger and Kostelecky 2011)
Eugenol	126 (107, 148)	694 (663, 726)	676 (618, 740)	268 (262, 275)	40 (Daenen *et al*., 2008)
Beta‐damascenone	3386 (2351, 4877)	3806 (3384, 4282)	4553 (3858, 5373)	4606 (3260, 6509)	150 (Praet *et al*., 2012)
Geraniol	455 (439, 471)	634 (538, 747)	658 (548, 789)	452 (437, 467)	4–40 (Schönberger and Kostelecky 2011)
Forest fruit milk beverages					
Benzyl alcohol	17 (9, 32)	1493 (1331, 1675)	530 (478, 586)	58 (19, 182)	900 000 (Meilgaard 1975)
Linalool	137 (90, 207)	399 (356, 448)	212 (105, 426)	160 (154, 165)	8–80 (Schönberger and Kostelecky 2011)
Methyl salicylate	nd	421 (173, 1024)	112 (106, 118)	nd	130 (Daenen *et al*., 2008)

nd, compound no detected.

Forest fruit milk beverages with *B. anomalus β*‐glucosidase contained more methyl salicylate (peppermint, wintergreen; Meilgaard 1975), benzyl alcohol and linalool than untreated beverages or beverages with AR2000. Compared to almond *β*‐glucosidase, the *B. anomalus* enzyme released more benzyl alcohol (Fig. 5). Considering that across *β*‐glucosidase enzyme treatments different aglycones exceeded their threshold concentration (Table 3), the resulting aroma of the beverages might be different. For this reason, beverages with different *β*‐glucosidases were subjected to double‐blind consumer preference tests to see if the aroma released by a specific enzyme was preferred.

### Preference tests of beers and milk beverages treated with *β*‐glucosidase enzymes

**Figure 6:**
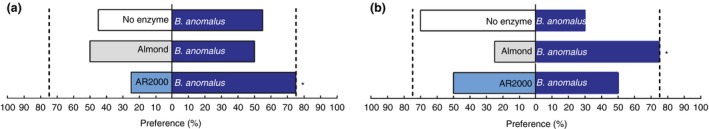
Double‐blind preference tests for cherry beers (a) and forest fruit milk beverages (b). A panel of 20 persons indicated whether they preferred the beverage with the *Brettanomyces anomalus* enzyme over beverages with AR2000, almond *β*‐glucosidase or without enzyme. Vertical dashed lines indicate the threshold to reach significant preferences. Among the panelists, there was a significant preference for cherry beers aromatized by *B. anomalus β*‐glucosidase over cherry beers with AR2000 and forest fruit milk beverages with *B. anomalus* instead of the almond *β*‐glucosidase.

In preference tests, the sensory panel indicated their preference for cherry beers with *B. anomalus* glucosidase over cherry beers with AR2000 (Fig. 6a). They described the beer with *B. anomalus β*‐glucosidase as fruitier and more cherry‐ and honey like, which corresponds to the increased release of eugenol (honey). Consumers also preferred forest fruit milk with the *B. anomalus* enzyme over milk with almond *β*‐glucosidase (Fig. 6b) which they attributed to a more spicy character of these beverages, as methyl salicylate exceeded its threshold concentration, while the aroma of almond treated milk was more neutral. Since spicy aromas usually are not associated with fruited milks, this also explains why 70% of the test panel preferred the untreated sample over the sample with the *B. anomalus* enzyme (although this preference was not significant).

## Discussion

Screening of 428 yeast strains for *β*‐glucosidase activity revealed strong activity in *Brettanomyces* strains. Although these yeasts are generally considered safe for food production, using them is often not possible as they are notorious for the formation of off‐flavours (Steensels *et al*. 2015). This study is the first to identify the *β*‐glucosidase‐encoding genes in *B. anomalus* and *B. bruxellensis*, and thus represents a first step towards using (enzymes of) these yeasts without their off‐flavours. After genome sequencing and identification of the *β*‐glucosidase‐encoding genes, we expressed the codon‐optimized genes in *E. coli* and isolated the resulting enzymes. Since the *B. anomalus β*‐glucosidase showed the highest enzymatic activity, we thoroughly characterized this protein by comparing it to commercially available *β*‐glucosidases (AR2000 and almond *β*‐glucosidase).

AR2000, *B. anomalus* and almond *β*‐glucosidase worked optimally at pH 4·5 and 58°C, pH 5·75 and 37°C and pH 5 and 50°C respectively. For the *A. niger* (AR2000) *β*‐glucosidase, optimal conditions between pH 4–4·5 and 55°C and 70°C, are described (Placzek 2014, accessed 12th November 2015). Although we found strongly reduced enzymatic activity at pH 3 and significant activity above pH 5, the packaging of AR2000 claims that it works optimally between pH 2·8 and 5, which might be a matrix effect. The results for almond *β*‐glucosidase do not agree with values described by Woodward and Wiseman (1982) (optimal pH of 5·6 on cellobiose). As the *B. anomalus* enzyme has a lower optimal temperature than AR2000 and almond glucosidase, it can be validated in ‘green’ food and biofuel applications as it does not require heating. However, although *k*_cat_ values indicated considerable activity at lower pH, the relative high pH optimum of the *B. anomalus β*‐glucosidase might be an issue for bioflavouring as most food products are slightly acidic. Hence, we tested the enzymes in applications that are characterized by a higher pH value like beer fermentations (pH 5·4 at the start of fermentation) and fruit milks (pH 5·5).

Cherry beers with *B. anomalus β*‐glucosidase contained significantly more benzyl alcohol and eugenol, which contribute to the typical aroma of cherry beers, which was preferred over samples with AR2000 (Schmid and Grosch 1986; Wen *et al*. 2014). Benzyl alcohol can be formed from benzyl glucosides (amygdalin and prunasin) which are present in cherry kernels (Chandra and Nair 1993). Also other aromatic compounds –mainly esters‐ differed between samples. Esters that are present as 1‐*O*‐glucosyl esters (like ethyl‐hydrocinnamate) can be released by transglucosidase activity of *β*‐glucosidases (Petersen and Matern 1999). Additionally, ester concentrations can differ because of stripping, where volatiles get lost because of CO_2_ purging of the fermentation medium (Haefliger and Jeckelmann 2013).

The aroma of forest fruit milks also differed depending on the *β*‐glucosidase used. Since the forest fruit was a mixture of different fruits, it was difficult to predict which aglycones were expected. However, all molecules listed in Fig. 5 (except p‐tolualdehyde) contribute to a characterizing strawberry aroma (Zabetakis and Holden 1997). The amount of glycosides hydrolysed was clearly influenced by the different pH and temperature optimum of the enzymes, as the almond enzyme released more aglycones in cherry beers, while the *B. anomalus β*‐glucosidase was much more active in fruited milk.

In conclusion, we identified, purified and characterized a novel *B. anomalus β*‐glucosidase that releases naturally present but hidden food flavours. Industrial application of this enzyme can be met by adding it during food production or by using the immobilization tag of the enzyme. Additionally, this study provides the first high‐quality genome sequence of *B. anomalus* and *B. bruxellensis*, opening the door for further exploration of their genomic properties.

## Conflict of Interest

No conflict of interest declared.

## Supporting information


**Figure S1** Synthetic DNA construct used to introduce the different glucosidase genes in the pET28 plasmid.

**Figure S2** Codon‐optimized *β*‐glucosidase genes of *Brettanomyces anomalus* (YV396) and *Brettanomyces bruxellensis* (YV397)

**Figure S3** Western blot (anti‐His) of cytoplasmic and inclusion body fraction before and after induction of *B. anomalus β*‐glucosidase synthesis.

**Figure S4** Enzymatic product formation by AR2000 (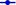
4·32*10^−3^ g l^−1^ and 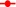
2·16*10^−6^ g l^−1^, Almond *β*‐glucosidase (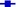
3·78*10^−2^ *μ*mol l^−1^ and 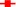
1·89*10^−2^ *μ*mol l^−1^) and *B. anomalus β*‐glucosidase (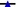
3·78*10^−2^ *μ*mol l^−1^ and 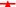
1·89*10^−2^ *μ*mol l^−1^) for cellobiose, amygdalin and salicin at pH 4·5 and 17°C, pH 5 and 17 or 37°C and pH 5·75 and 37°C at different incubation times.

**Figure S5** Temperature program used during GC‐MS analysis.

**Figure S6** Alignment of the amino acid sequences of the GH3 *β*‐glucosidase enzymes of *Brettanomyces bruxellensis, Brettanomyces anomalus* and *Kluyveromyces marxianus*.

**Figure S7** Absolute enzymatic activity of the heat‐treated glucosidases.

**Figure S8** Michaelis–Menten plots for AR2000 (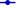
4·32*10^−3^ g l^−1^ and 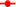
2·16*10^−6^ g l^−1^, Almond *β*‐glucosidase (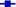
3·78*10^−2^ *μ*mol l^−1^ and 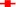
1·89*10^−2^ *μ*mol l^−1^) and *B. anomalus β*‐glucosidase (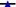
3·78*10^−2^ *μ*mol l^−1^ and 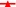
1·89*10^−2^ *μ*mol l^−1^), almond and *B. anomalus β*‐glucosidase for cellobiose, amygdalin and salicin at pH 4·5 17°C and pH 5·75 37°C.

**Table S1** *β*‐glucosidase activity of screened yeast strains.

**Table S2** Composition of agar media used for yeast screening.

**Table S3** Main results from the qualitative screening of 428 yeast strains for *β*‐glucosidase activity on various agar media.
