# Supplementary material for: Characterization of the recombinant Brettanomyces anomalus β‐glucosidase and its potential for bioflavouring
Source: J Appl Microbiol. 2016 Jul 27;121(3):721–33. doi: 10.1111/jam.13200 (PMC6680314; doi:10.1111/jam.13200)
Supplement: Supplementary file 1 — Figure S1 Synthetic DNA construct used to introduce the different glucosidase genes in the pET28 plasmid. [file JAM-121-721-s001.pdf]

Figure S1

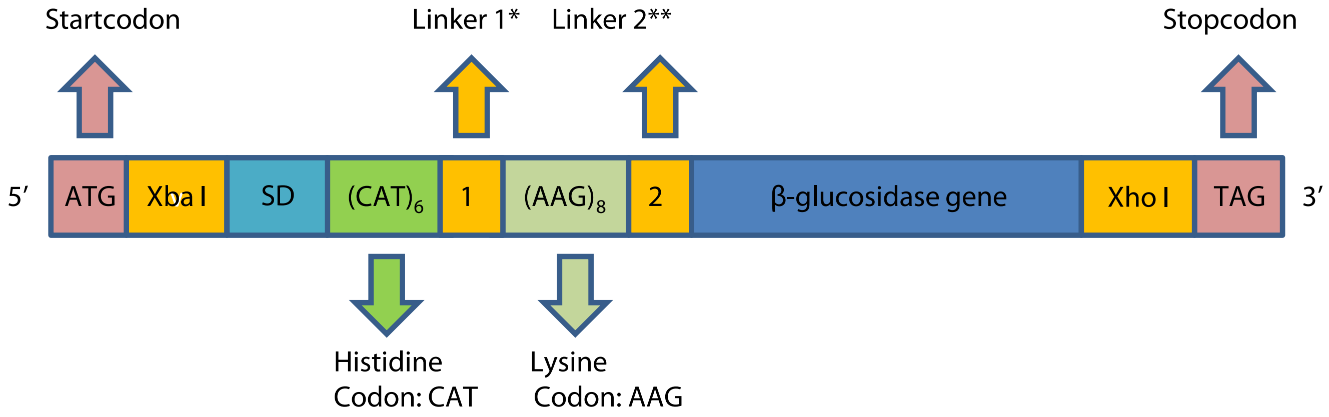

* Linker 1 = cutting place for XbaI and factor Xa protease
** Linker 2= cutting place for trombine and XhoI
