# Supplementary material for: Characterization of the recombinant Brettanomyces anomalus β‐glucosidase and its potential for bioflavouring
Source: J Appl Microbiol. 2016 Jul 27;121(3):721–33. doi: 10.1111/jam.13200 (PMC6680314; doi:10.1111/jam.13200)
Supplement: Supplementary file 2 — Figure S2 Codon‐optimized β‐glucosidase genes of Brettanomyces anomalus (YV396) and Brettanomyces bruxellensis (YV397) [file JAM-121-721-s002.pdf]

Figure S2

Codon optimized YV396 β-glucosidase gene fragment

ATGTCTAGAAGGAGGCATCACCATCATCACCACAGCAGCATTGAAGGTCGCAAGAAGAAGAAAAAGAAAAAAAA
ATCCAGCGGTCTGGTTCCGCGTGGTAGCCATACCTTTGATGTGGATAGCATTCTGGGCCAGCTGACCACCGAAGA
GAAAGTTAGCCTGATTGCAGCAGTTGATAATTGGCATACCAAAGAAATTAAACGCCTGGATATTCCGGCAATTCGT
GTTAGTGATGGTCCGAATGGTATTCGTGGCACCCGCTTTTTTAACGGTGTTCCGAGCGCATGTTTTCCGAATGGCA
CCGGTCTGGCAGCAACATTTGATGCCGATCTGCTGGAAAAAGCCGGTGAACTGATGAGTATTGAAGCCGAACATA
AAAATGCCCAGGTTATTCTGGGTCCGACCACCAATATTCAGCGTGGTCCGCTGGGTGGTCGTGGTTTTGAAAGCTT
TAGCGAAGATCCGTTTCTGAGCGGTATTTGTACCGCAAGCATTGTTAATGGCATTCAGAAAAGCGGTAAAATTGGT
GCGACCGTGAAACATTTTGTTTGCAATGATCTGGAAGATCAGCGCTTTAGCAGCAATAGCGTTCTGACCCAGCGTG
CACTGCGTGAAATTTATCTGGAACCGTTTCGTCTGGCAGTTAAACTGGCAGATCCGAAATGTTTTATGACCAGCTAT
AACAAAGTGAACGATGAACATTGCAGCCAGAACTATCATCTGATTGAAGAAATTCTGCGTGGCGAATGGAATTGG
AAAGGTATGATTATGAGCGATTGGTTTGGCACCTATAGCACCGCAGCAGCCCTGAAACATGGTATTGATATTGAAT
TTCCGGGTCCGACAAAATTTCGTCGTTGGGAAGTTGTTAAACATCTGCTGCAGAGCAAAGAAGCAGATCTGAAAG
AAGAGGATATTGATAATCGTTGCCGCAATGTTCTGAAACTGATCAAATTTGTTGTTGACACCCGTGGTAATGGTCC
GTATCCGACAGCCGAAGATACCAAAAATGATACACCGGAAACCAGCGCAAAACTGCGTAATCTGGCAGCCCAGG
GTATTGTTCTGCTGAAAAATGATCGTGGTGTTCTGCCGCTGAGTAAAGATAAAAGCACCGTTGTTATTGGTCCGAA
CGGCAAAGCACTGAATACAATTAGCGGTGGTGGTAGCGCAAGCATGCGTCCGTATCATGTTGTTACCCCGTATGA
TGGTATTAAAAGCAAAGTGGGTAAAGTGGATTACACCGTTGGTTGCTATTGTGACAAAGCTCTGAAAAACCTGTTT
GAATTCATGACCAACGATCTGGACAAAAGCAAAAAAGGTGTTAAAGCCACCTTCTACACCAAAGCCTTTGAAAATC
GTGATGCAAGCGATAAACCGATCGATGAAATGATTGTTGATAGCAGCTTTGTGACCCTGTTCGATTATAGCAATCC
GGCAGTGGATAGCGAAAAAAAACTGTTCTATGTGGATTTCGAGGGCTATTATACTCCGGATGCAACCGCAGATTA
CAAATTTGGTTGTCAGGTTTTTGGCACCGCACTGGTTTATGTTGATGGTAAACTGCTGATCGATAACAAAACCAGC
CAGACCAAAGGCACCTTTTGTTTTAGCAGCGGCACCGTTGAAGAAACCGCAGTTACCCATCTGGAAGCAGGTCAT
AGCTATAAAATCAAAGTTGAATTTGGCAGCGGCATCACCAGCAAAATTAGCAGCGATTTTGGTAGTGGTGGTCTG
CAGGTTGGTATTACCAAAGTTATTGATCCGGAAATCGAAGTGGAACATGCCGCAGAACTGGCAAAAAGCCATGAT
AATGTTATTCTGTGCATTGGCCTGAATGGTGAATGGGAAAGCGAAGGTTATGATCGTGCAGATATGACCCTGCCT
GGTAAAACCAATGACCTGGTTAGCGCAGTTCTGAAAGCAAATCCGAATACCGTTATTGTTAATCAGAGCGGTACAC
CGGTTGAAATGCCGTGGCTGGGTGAAAGCCATACCCTGCTGCAGGCCTGGTATGGTGGTAATGAAATGGGTGAT
GCACTGGCCGATATTCTGTTTGGTGATGCGGTGCCGAGCGGTAAACTGAGCCTGAGCTGGCCGTTTAAAAACCAG
GATAATCCGGCATATCTGAACTTTAGCACCGAAATGGGTCGTGTTCTGTATGGTGAAGATGTTTTTGTGGGCTATC
GCTATTATGAAAAACTGCAGCGTCGTGTTGCATTTCCGTTTGGTTATGGTCTGAGCTATACCACCTTTAAATTCGAT
GACCTGAAAGTGAGCAGCGATAATACCAATGTTACCGTTAGCTTTACCGTGAAAAATACCGGTGATAAATACACCG
CAAAAGAAGTTACCCAGCTGTATATTTCTGCAGTTGAAAGCAGCGTTACCCGTCCGGTTAAAGAACTGAAAGCATT
TGCAAAACCGGAACTGAAACCGGGTGAAAGTAAAACCGTTTCATTTAATCTGAGCCTGGAAGATGCCTGCAGCTTT
TTTGATGAATATCGCAATAAATGGTGTCTGGAAGCCGGTAAATATGAAGCACAGGTGGGTAGCAGCAGTGATGAT
ATTCACCTGATTGGCGAATTTGATGTTGCCAAAACCGTGTATTTTGTTCGTAGCGCATAACTCGAGTTTAG

Legend

Lysine tag Histidine tag SD sequence Xba I restriction site β-glucosidase gene xHoI restriction site

Start - and stopcodon Linker 1 and 2

Codon optimized YV397 β-glucosidase gene fragment

ATGTCTAGACATCACCATCATCACCACAGCAGCATTGAAGGTCGCAAGAAGAAGAAAAAGAAAAAAAAAATCCAGC
GGTCTGGTTCCGCGTGGTAGCCATATTTTTGATGTTGAAAATACCCTGCGTGATCTGACCACCGAAGAGAAAGTTA
GCCTGCTGAGCGCAAGCGATTTTTGGCATACCAGCAGTATTGAACGTCTGAACATTCCGTTTATTCGTGTTAGTGAT
GGTCCGAATGGTATTCGTGGCACCAAATTTTTCAATGGTGTTCCGAGCGCATGTTTTCCGAATGGCACCGGTCTGG
CAAGCACCTTTGATTGTGATCTGCTGGAAGAAATTGGTGAACTGATGGCAGTTGAAGCCAAACATAAAGGTGCAC
AGATTATTCTGGGTCCGACCACCAATATTCTGCGTGGTCCGCTGGGTGGTCGTGGTTTTGAAAGCTTTAGCGAAGA
TCCGGTTCTGAGCGGTCTGTGTACCGCAGCAATTGTTAAAGGTATTCAGAATGATGGTCGTATTTGCGCCACCGTT
AAACATTTTGTTTGCAACGATCTGGAACATGAACGCCTGAGCAGCAATAGCGTTGTTAGCGAACGTGCACTGCGT
GAAATTTATCTGGAACCGTTTCGTATTGCAGTTCAGCTGGCAAATCCGATTTGTATTATGACCGCCTATAACAAAGT
GAACGGTATTCATTGCAGCGAGAATTACCAGCTGATTGAAAACATTCTGCGCAAAGAATGGAATTGGGATGGTCT
GCTGATGAGCGATTGGTTTGGCACCTATAGCACCCTGAATAGCCTGAAACATGGCATTGATATTGAATTTCCGGGT
CCGAGCCAGTTTCGTCGTTGGGATACCATTAAACATCTGCTGCAGAGCAAAGCCGATAATCTGAAACAGACCGATA
TTGATAATCACTGCCGTCATATTCTGAAAGTGATCAAAAGCCTGATCGAAAGCAATGGCACCACCCTGTTTAGCAA
AGTTGAAGATAGTCTGAACGATAAACCGGAAACCAGCGAAAAACTGCGTCGTGCAGCAGCAGAAGGTATTGTTCT
GCTGAAAAATGAACGTAAAGTTCTGCCGCTGCTGAAAGAAACACCGGTTCTGGTTATTGGTCCGAACGCAATTAG
TCTGAATACATATAGCGGTGGTGGTAGCGCAAGCCTGACCCCGTATCATATTGTTACACCGCTGCAGGGCATTAAA
AAAAAAGCAAGCAAAGTGGAATTTACCATCGGTGCACATAGCCATAAAGCACTGGGTGGCCTGTTTGAAAAAATG
ACCAATGATCTGGAAAAATTCGAAAATGGTGTGCGTGCACGTTTTTATACCAAACCGCGTGAAAAACGCCTGAAA
GAAGATAAACCGATTGACGAAATGATCATCAAAAACAGCTATGTGACCCTGTTCGATTATACCAATCCGGCAGTTA
ATCAAGAAAGCAAACTGTTCTATGCCGATTTCGAAGGTTATTACACCCCGACCGAAAGTGGCGATTATCAGATTGG
TTGTCAGGTTGCAGGCACCGCAATTGTTTATATTGATGACAAAATTCTGATCGATAACAAAACCAAACAGACCAAA
GGCACCTTTTGCTTTAGCGGTGGCACAATTGAAGAAACCGCATGCATTTATATGCAGGCCTATCAGAAATATCGCA
TCAAAGTAGAATTTGGCAGCGGTATTACGAGCAAAATCTATACCAATTTTGGTGCCGGTGGTCTGCAGGTTGGTAT
TACCAAAGTTATTGATCCGCAGAAAGAAATCAAAAAAGCAGCCAGCCTGGCAGCAAGCTATCAGAATGTTATTCT
GTGTATTGGTCTGAACAGCGAATGGGAAAGTGAAGGTTATGATCGCGAAGATATGAAACTGCCTGGTCGTACCGA
TGATCTGGTTCGTGCAGTTATTCAGGCCAATCCGAATACCGTGGTTATTAATCAGAGCGGTACACCGGTTGAAATG
CCGTGGCTGGATCAGTGTGATACCCTGCTGCAAACCTGGTATGGCGGTGATGAACTGGGTGATGCAGTTGCAGAT
ATTCTGTATGGTGATACCATTCCGTGTGGTAAACTGCCGTTTAGCTGGCCTGCAAAAAATGAAGATAATCCGAGCT
TTCTGAACTTCCGCACCGAAAAAGGTCGTGTGCTGTATGGCGAAGATGTTTATGTTGGTTATCGCTACTATGAGAA
ACTGCATCGTAATGTTGCATTTCCGTTTGGTTATGGTCTGAGCTATACCCAGTTTGAATATCAGAACCTGAAAGTAA
GCAGCGATAGCAATAATCTGATCCTGAGCTTTGAGATTAAAAACATTGGTCAGTACGCAGGCAAAGAAACAGCAC
AGGTGTATATTAGCAGCCTGGGTCCTACCATTAGCCGTCCGAGCAAAGAACTGAAAGCATTTATGAAAACCACCCT
GGAACCGGGTGAAAGCAAAGTTATGAATTTTCGCCTGAAATTCAAAGAAATCTGCAGCTATTATGATGAGTATCA
GAAAATGTGGTGTCTGGAAAGCGGTAAATATCTGGCACTGGTTGGTGCAAGCAGCATGAATATTAGCCTGACCGG
TAGCTTTGATATTCTGGAAACCACCTATTTTGAGAAACGCTGCTAACTCGAGGCACCACCACCACCACCACTGA

Legend

Lysine tag Histidine tag SD sequence Xba I restriction site β-glucosidase gene xHoI restriction site

Start - and stopcodon Linker 1 and 2
