# Supplementary figures and images for: Characterization of the recombinant Brettanomyces anomalus β‐glucosidase and its potential for bioflavouring
Source: J Appl Microbiol. 2016 Jul 27;121(3):721–33. doi: 10.1111/jam.13200 (PMC6680314; doi:10.1111/jam.13200)

Figure S3

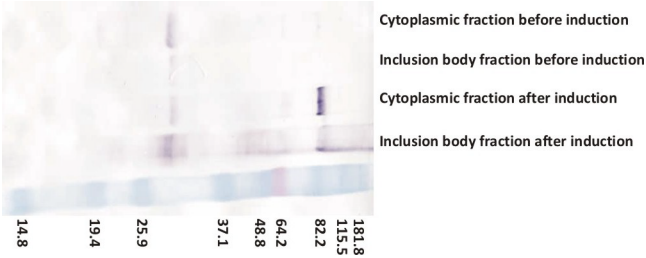

Supplement: Supplementary file 3 — Figure S3 Western blot (anti‐His) of cytoplasmic and inclusion body fraction before and after induction of B. anomalus β‐glucosidase synthesis. [file JAM-121-721-s003.pdf]

Figure S4

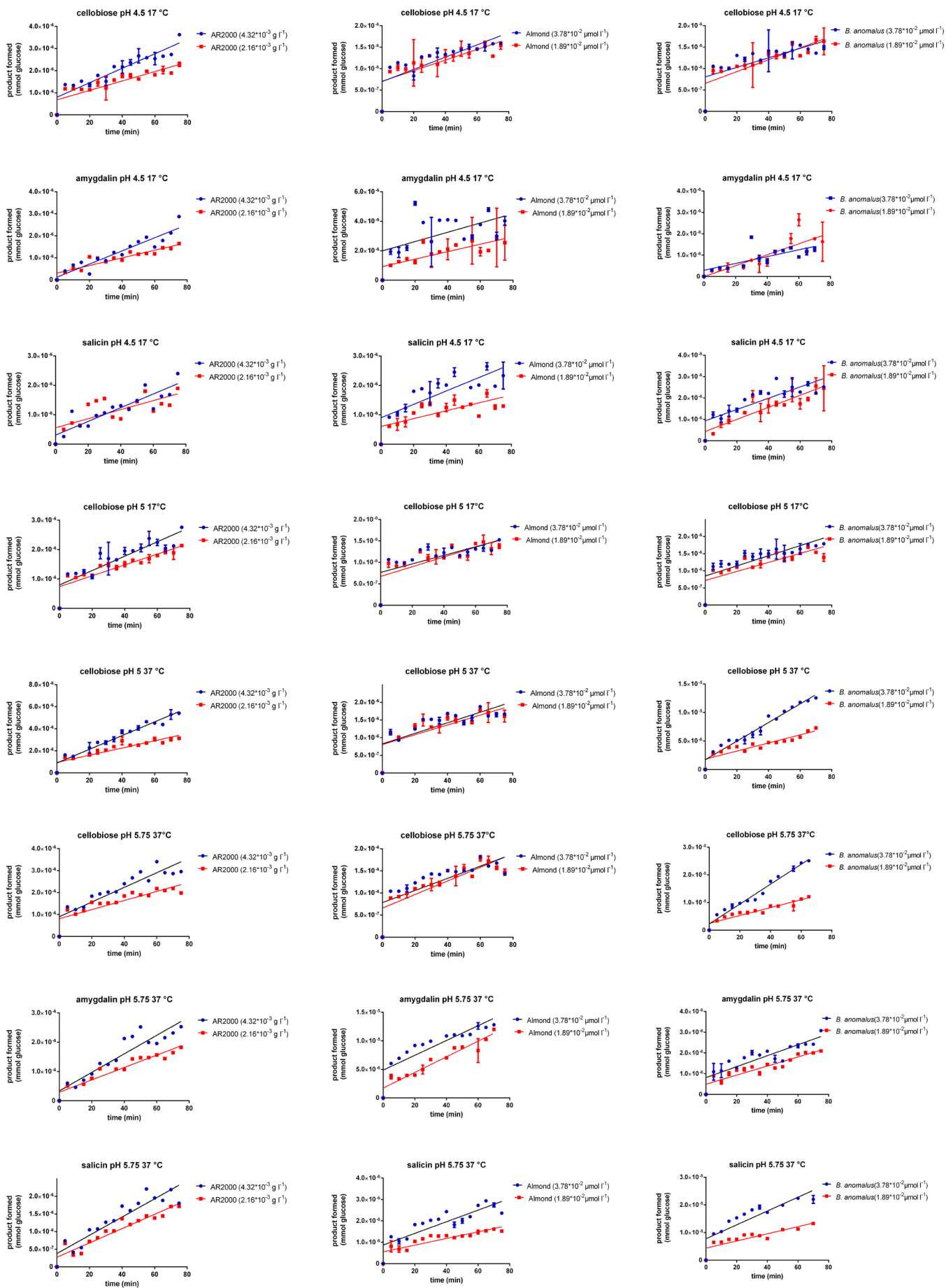

Supplement: Supplementary file 4 — Figure S4 Enzymatic product formation by AR2000 (4·32*10−3 g l−1 and 2·16*10−6 g l−1, Almond β‐glucosidase (3·78*10−2 μmol l−1 and 1·89*10−2 μmol l−1) and B. anomalus β‐glucosidase (3·78*10−2 μmol l−1 and 1·89*10−2 μmol l−1) for cellobiose, amygdalin and salicin at pH 4·5 and 17°C, pH 5 and 17 or 37°C and pH 5·75 and 37°C at different incubation times. [file JAM-121-721-s004.pdf]

Figure S5

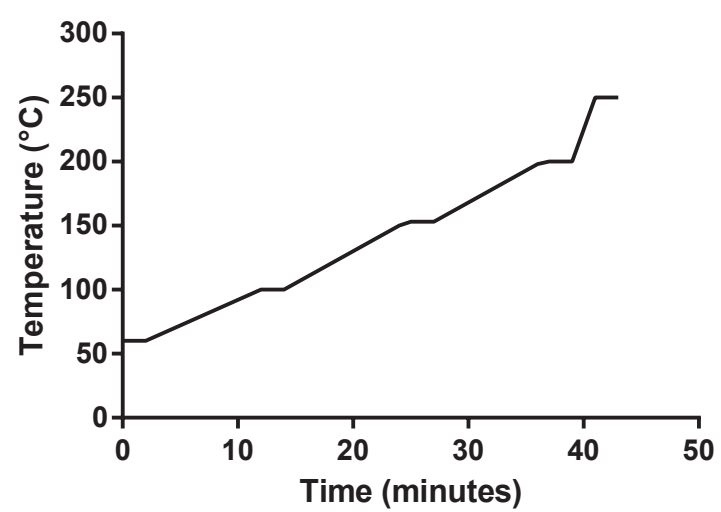

Supplement: Supplementary file 5 — Figure S5 Temperature program used during GC‐MS analysis. [file JAM-121-721-s005.pdf]

Figure S6

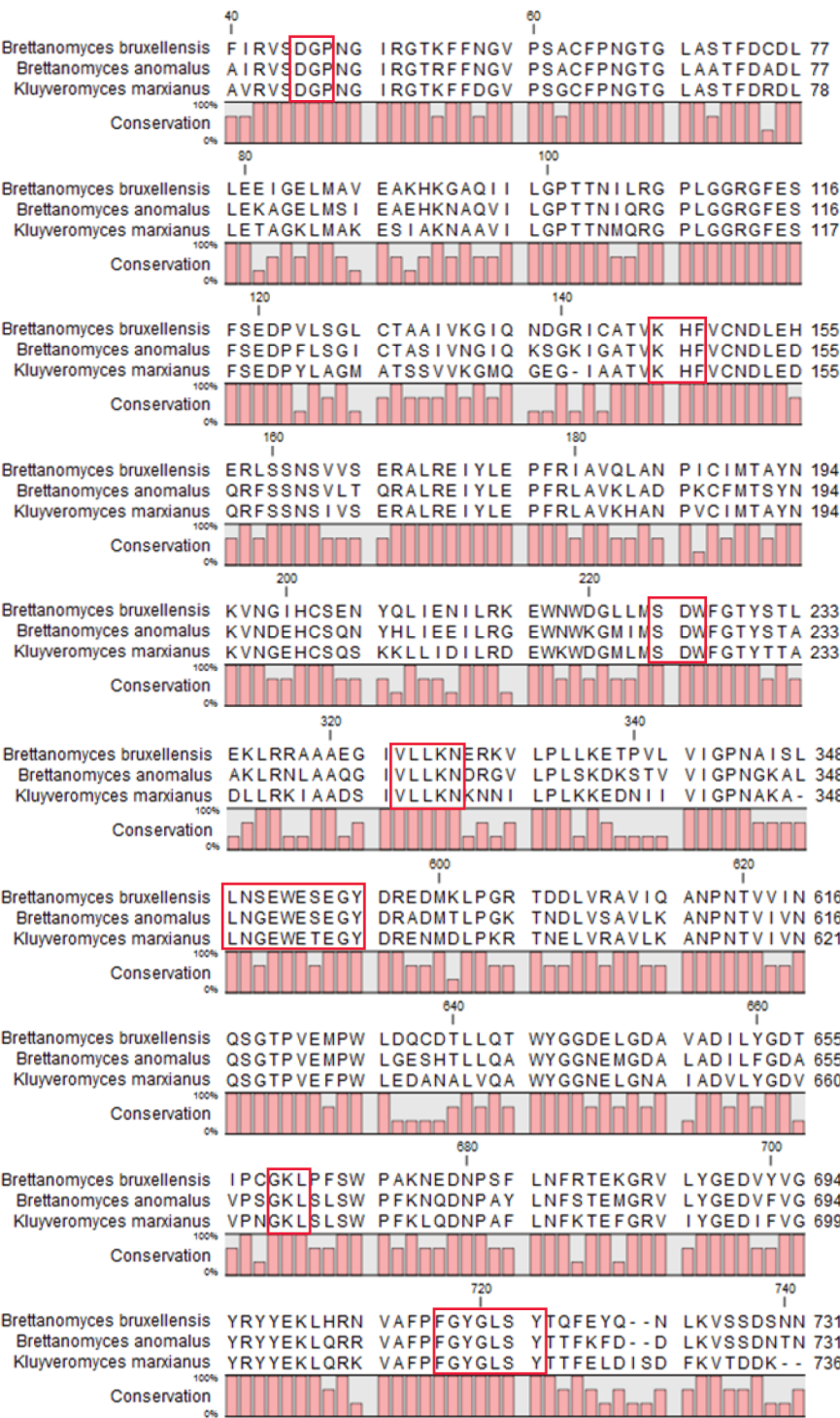

Supplement: Supplementary file 6 — Figure S6 Alignment of the amino acid sequences of the GH3 β‐glucosidase enzymes of Brettanomyces bruxellensis, Brettanomyces anomalus and Kluyveromyces marxianus. [file JAM-121-721-s006.pdf]

Figure S7

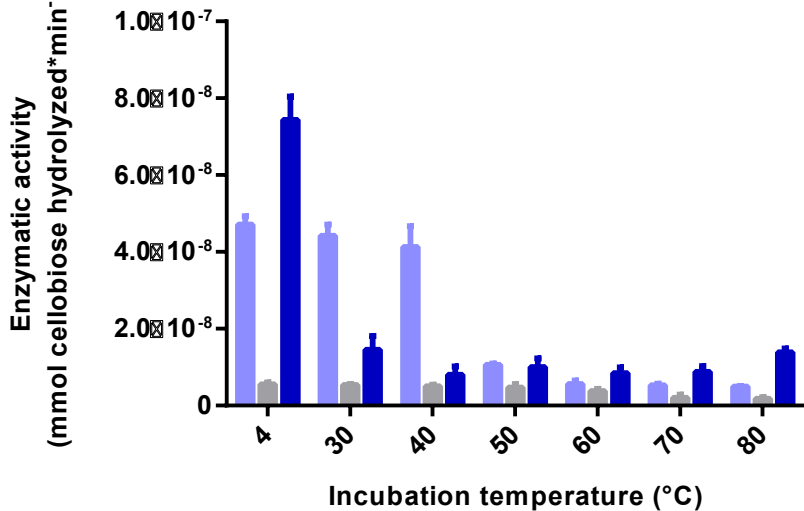

Supplement: Supplementary file 7 — Figure S7 Absolute enzymatic activity of the heat‐treated glucosidases. [file JAM-121-721-s007.pdf]

Figure S8

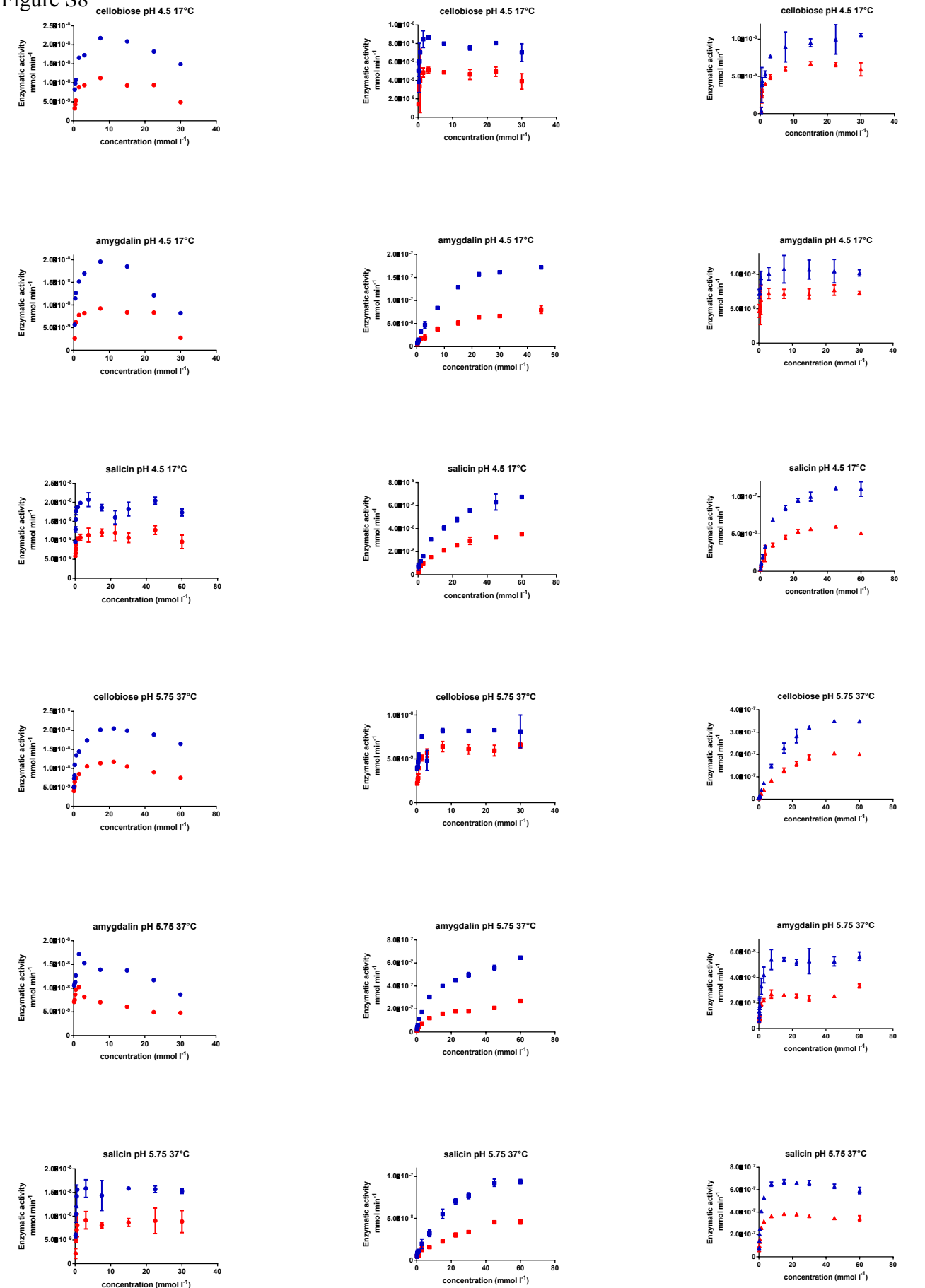

Supplement: Supplementary file 8 — Figure S8 Michaelis–Menten plots for AR2000 (4·32*10−3 g l−1 and 2·16*10−6 g l−1, Almond β‐glucosidase (3·78*10−2 μmol l−1 and 1·89*10−2 μmol l−1) and B. anomalus β‐glucosidase (3·78*10−2 μmol l−1 and 1·89*10−2 μmol l−1), almond and B. anomalus β‐glucosidase for cellobiose, amygdalin and salicin at pH 4·5 17°C and pH 5·75 37°C. [file JAM-121-721-s008.pdf]
