# Supplementary material for: Characterization of the recombinant Brettanomyces anomalus β‐glucosidase and its potential for bioflavouring
Source: J Appl Microbiol. 2016 Jul 27;121(3):721–33. doi: 10.1111/jam.13200 (PMC6680314; doi:10.1111/jam.13200)
Supplement: Supplementary file 9 — Table S1 β‐glucosidase activity of screened yeast strains. [file JAM-121-721-s009.pdf]

Table S1

Yeast number	Type	Number of strains	Number of strains growing on all β-glucoside media
**BY4741**	**Lab (Haploid S288c)**	1	0
**YV1 -YV127**	**Industrial (Ale)**	127	37
**YV128-YV146**	**Industrial (lager)**	19	12
**YV147-YV217**	**Industrial (Wine)**	71	49
**YV218-YV230**	**Industrial (Sake)**	13	4
**YV231-YV241**	**Industrial (Spirits)**	11	5
**YV242-YV248**	**Industrial (Bioethanol)**	7	3
**YV249-YV258**	**Industrial (Bakery)**	10	8
**YV259-YV311**	**Feral**	53	23
**YV312-YV427**	**Non-*Saccharomyces***	116	55
**Total**		428	196
