# Supplementary material for: Characterization of the recombinant Brettanomyces anomalus β‐glucosidase and its potential for bioflavouring
Source: J Appl Microbiol. 2016 Jul 27;121(3):721–33. doi: 10.1111/jam.13200 (PMC6680314; doi:10.1111/jam.13200)
Supplement: Supplementary file 10 — Table S2 Composition of agar media used for yeast screening. [file JAM-121-721-s010.pdf]

Table S2

	Culture media				
	**YPD**	**YPEtOH**	**YPArbutin**	**YPCellobiose**	**YPSalicin**
**Agar**	2%	2%	2%	2%	2%
**Yeast extract**	1%	1%			
**Bactopeptone**	2%	2%			
**YNB**			0.17%	0.17%	0.17%
**AA mix with $(NH_4)_2SO_4$**			0.5%	0.5%	0.5%
**D-glucose**	2%				
**EtOH**		2%			
**Arbutin**			0.5%		
**Cellobiose**				0.5%	
**Salicin**					0.5%
