# Supplementary material for: Characterization of the recombinant Brettanomyces anomalus β‐glucosidase and its potential for bioflavouring
Source: J Appl Microbiol. 2016 Jul 27;121(3):721–33. doi: 10.1111/jam.13200 (PMC6680314; doi:10.1111/jam.13200)
Supplement: Supplementary file 11 — Table S3 Main results from the qualitative screening of 428 yeast strains for β‐glucosidase activity on various agar media. [file JAM-121-721-s011.pdf]

Table S3

	YPD	YPEtOH	YPArbutin	YPCellobiose	YPSalicin
**BY4741 (β-glucosidase negative)**	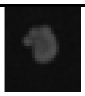	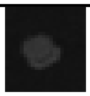	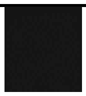	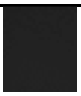	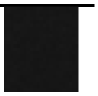
**YV396 (*B. anomalus*)**	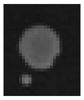	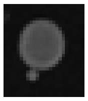	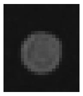	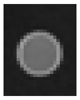	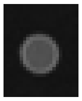
**YV397 (*B.* bruxellensis)**	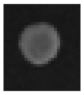	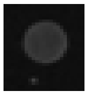	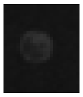	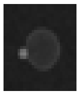	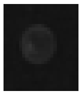
**YV404 (*K. marxianus)***	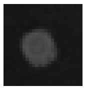	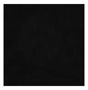	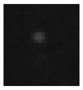	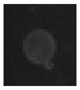	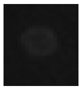
**YV15 (*S. cerevisiae*)**	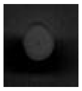	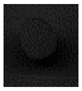	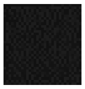	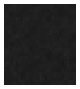	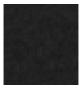
